# Gingival and alveolar mucosal reactive hyperplastic lesions: a retrospective clinical and histological study of 996 cases

**DOI:** 10.4317/medoral.25766

**Published:** 2023-01-15

**Authors:** Rafaela Carriço Porto Baesso, Rebeca Souza Azevedo, Bruna Lavinas Sayed Picciani, Fábio Ramoa Pires

**Affiliations:** 1 DDS, MSc. Postgraduate Program in Dentistry, Health Institute of Nova Friburgo (PPGO-ISNF), Fluminense Federal University (UFF), Nova Friburgo/RJ, Brazil; 2 PhD Student. Postgraduate Program in Dentistry, Estácio de Sá University, Rio de Janeiro/RJ, Brazil; 3 PhD, Professor. Oral Pathology, School of Dentistry, Fluminense Federal University, Nova Friburgo/RJ, Brazil; 4 PhD, Professor. Postgraduate Program in Dentistry, Health Institute of Nova Friburgo (PPGO-ISNF), Fluminense Federal University (UFF), Nova Friburgo/RJ, Brazil; 5 PhD, Professor. Oral Pathology, Dental School, Rio de Janeiro State University, Rio de Janeiro/RJ, Brazil; 6 PhD, Professor. Postgraduate Program in Dentistry, Estácio de Sá University, Rio de Janeiro/RJ, Brazil

## Abstract

**Background:**

gingival/alveolar mucosal reactive hyperplastic lesions (GRHL), including fibrous hyperplasia (FH), pyogenic granuloma (PG), peripheral ossifying fibroma (POF) and peripheral giant cell lesion (PGCL), are a common group of oral diseases. The aim of the present study was to access the frequency and distribution of the clinical and histological features of these disorders in a Brazilian population.

**Material and Methods:**

all specimens diagnosed as GRHL in three Oral Pathology laboratories were selected for the study. Clinical information was retrieved from the laboratory biopsy forms and hematoxylin and eosin stained histological slides were reviewed for analysis of the histological characteristics.

**Results:**

final sample was composed of 996 specimens, including 463 FH (47%), 280 PG (28%), 183 POF (18%) and 70 PGCL (7%). Females were more affected by FH, PG, and POF, and most cases affected adults with mean ages ranging from 40 to 53 years. FH, PG, and POF were more common in the upper gingiva/alveolar mucosa. Most PG, POF and PGCL were pedunculated, in contrast with FH (*p*<0.001). PG, FH and POF were mostly red or normal mucosal in color, while PGCL were mostly red/purple (*p*<0.001). PGCL were larger, followed by POF, FH and PG (*p*<0.001). Some histological features were characteristically found in some conditions, but they were also encountered in other lesions with variable frequencies.

**Conclusions:**

Oral medicine specialists, oral pathologists and periodontists are usually the professionals in contact with patients presenting GRHL and it is of upmost relevance that they should be familiarized with their clinical and histological profile.

** Key words:**Gingiva, lesions, reactive, hyperplastic.

## Introduction

Gingival and alveolar mucosal swellings are very common in routine Oral Medicine practice and comprise a variety of inflammatory, reactive, infectious and neoplastic conditions (1-3). Gingival/alveolar mucosal reactive hyperplastic lesions (GRHL) are one of the most common group of conditions that affect the oral cavity, representing from 3.6% to 10% of all diagnosis rendered in Oral Pathology laboratories (4-10). In this specific group, fibrous hyperplasia/fibroma (FH), pyogenic granuloma (PG), peripheral ossifying fibroma (POF) and peripheral giant cell lesion (PGCL) are the most common conditions (1,2,5,11,12).

It is accepted that these disorders arise from the periodontal tissues and that their etiology is directly associated with chronic local irritation due to deficient local hygiene and consequent biofilm and calculus accumulation, ill-fitting restorations, prosthetic and orthodontic appliances, occlusal imbalance/trauma, foreign bodies, iatrogenic factors, and some specific systemic factors (such as hormonal changes associated with pregnancy) (1,3). Although the etiological factors seem to be similar, tissue responses in each of these lesions produce different clinical and histological alterations.

Some studies have shown the differences on frequency and distribution of clinical and histological parameters from this group of lesions in different populations. Thus, the aim of the present study was to access the frequency of FH, PG, POF and PGCL diagnosed in a Brazilian population and to establish the clinical and histological profile of these conditions in this specific population.

## Material and Methods

This was a retrospective observational study and it was approved by the Ethics in Research Committee, Antônio Pedro University Hospital, Fluminense Federal University (protocol 2.972.573) and conducted in accordance with the Helsinki Declaration as revised in 2013. All specimens registered and diagnosed as GRHL, including FH, PG, POF and PGCL, in the Oral Pathology laboratories from the Fluminense Federal University (Nova Friburgo/RJ, from 2010 to 2018), Rio de Janeiro State University (Rio de Janeiro/RJ, from 2005 to 2018) and Estácio de Sá University (Rio de Janeiro/RJ, from 1999 to 2018) were retrieved from the laboratories registries. After this initial selection, all hematoxylin and eosin (HE) stained histological slides were reviewed under light microscopy for diagnosis confirmation. Specimens without conclusive diagnosis and without available clinical information in the laboratories files were excluded from the final sample. FH group included lesions presenting a connective tissue composed by a fibrous collagenous component associated or not with an inflammatory infiltrate of variable intensity (grouping all lesions diagnosed as fibrous hyperplasia and inflammatory fibrous hyperplasia). Specimens diagnosed as compatible with gingivitis, drug-related gingival enlargements, palatal fibromatosis, and hereditary gingival fibromatosis were not included in the final sample.

The laboratory charts were reviewed and information about age, gender, type of biopsy (excisional or incisional), site (upper or lower; anterior - incisives and canines - or posterior - premolars and molars - gingiva/alveolar mucosa), associated teeth, insertion (sessile or pedunculated), color (normal mucosa, red, purple/blue, brown), size of the gross specimens (in cubic milimeters, exclusive for excisional biopsies), time of onset (in months) and final histological diagnosis were retrieved from the specimens.

Five µm HE stained histological sections from all cases were reviewed under light microscopy and additional sections were obtained from the paraffin blocks when necessary. Histological analysis was always performed by two professionals and included the following parameters: presence of ulceration; type of connective tissue (loose, myxoid or dense); inflammatory infiltrate (predominantly chronic or acute); and presence of vascular proliferation, fibroblast proliferation, mineralized tissue (compatible with bone, cementum or distrophic calcification), multinucleated giant cells, hemorrhage, and bacterial colonies. The intensity of the inflammatory infiltrate was also classified as focal (inflammatory cells representing up to 5% from all connective tissue cells and mostly in the subepithelial region), mild (inflammatory cells representing 5 to 50% of the connective tissue cells) and moderate/intense (inflammatory cells representing more than 50% of the connective tissue cells); the percent of inflammatory cells was estimated based on the analysis of the connective tissue from each specimen in high magnification (400x) under light microscopy.

All clinical and histological information were included in a data bank designed specifically for the study. Statistical analysis was performed by the Statistical Package for Social Sciences (SPSS, version 22.0), and included a descriptive analysis, and a comparison of the studied variables among the four diagnostic groups by the use of the chi-square (Pearson), ANOVA and Spearman tests with a significance level of 5% (*p*<0.05).

## Results

**Figure 1:**
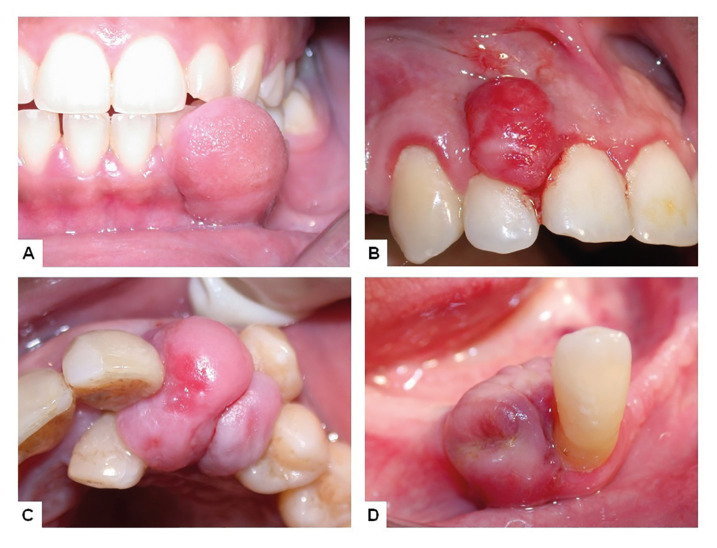
Clinical presentation of fibrous hyperplasia (A), pyogenic granuloma (B), peripheral ossifying fibroma (C) and peripheral giant cell lesion (D).

The final sample was composed by 996 specimens, including 463 FH (47%), 280 PG (28%), 183 POF (18%) and 70 PGCL (7%) (Fig. 1). Females represented 67%, 70% and 66% of the patients affected by, respectively, FH, PG, and POF, in contrast to 49% of the PGCL affected patients (*p*=0.008). Age of the patients ranged from 3 to 95 years old, with mean ages ranging from 40 to 53 years, depending on the final diagnosis. Excisional biopsies were performed in 92% of the cases (Table 1).

**Table 1:**
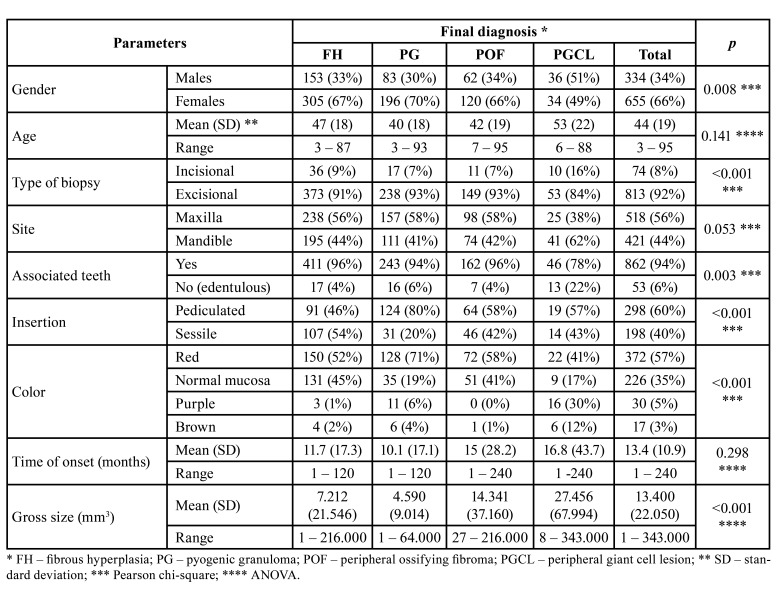
Distribution of the clinical parameters by final diagnosis.

**Figure 2:**
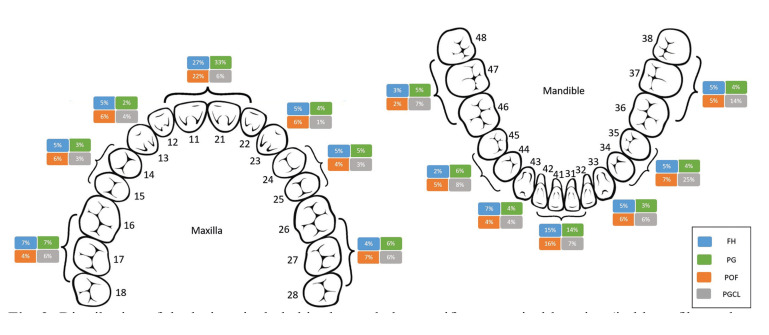
Distribution of the lesions included in the study by specific anatomical location (in blue - fibrous hyperplasia; in green - pyogenic granuloma; in orange - peripheral ossifying fibroma; in grey - peripheral giant cell lesion).

Site distribution showed that FH, PG, and POF were more common in the upper gingiva/alveolar mucosa, in contrast with PGCL. The upper anterior region was the most affected site (275 lesions), followed by the lower anterior region [191], the upper posterior region [166] and the lower posterior region [155]; site was desbribed solely as upper or lower gingiva/alveolar mucosa in, respectively, 77 and 75 cases. The most frequently teeth associated with FH, PG and POF were the upper central incisors, in contrast to lower premolars in PGCL. Edentulous areas were more frequently associated with PGCL (*p*=0.003) (Table 1 and Fig. 2).

Most PG, POF and PGCL were pedunculated, in contrast with FH (*p*<0.001). PG, FH and POF were mostly red or normal mucosal in color, while PGCL were mostly red or purple (*p*<0.001). Time of onset was higher for POF and PGCL, but the differences to FH and PG were not statistically significant. Gross size of the specimens submitted to the laboratories showed that PGCL were larger, followed by POF, FH and PG (*p*<0.001) (Table 1).

**Figure 3:**
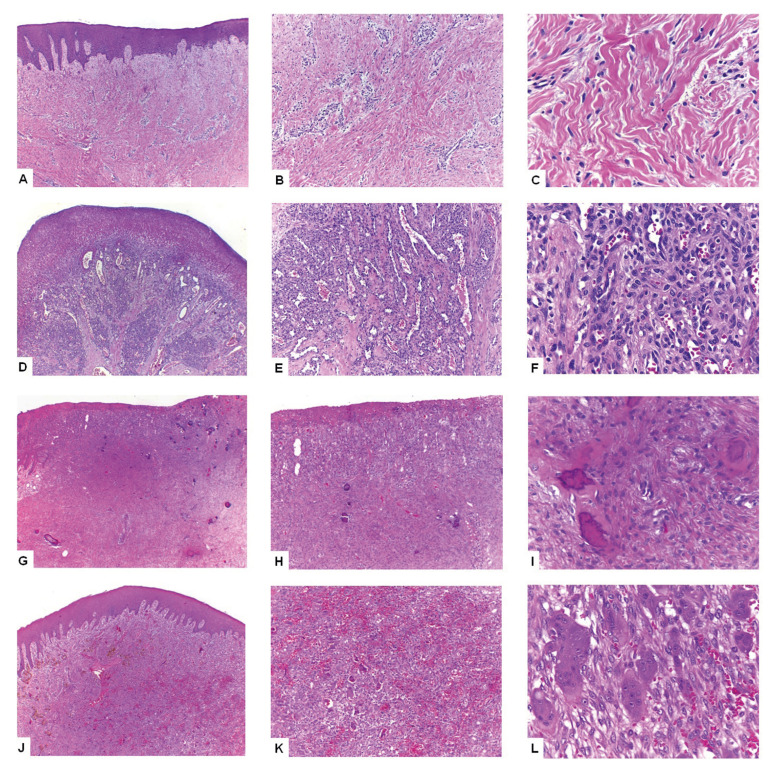
Hematoxylin and eosin (HE)-stained sections from the studied lesions. Inflammatory fibrous hyperplasia showing a fibrous proliferation covered by a stratified squamous epithelium (A, HE 40x) and details of the fibrous component permeated by a mild chronic inflammatory infiltrate (B, HE 100x and C, HE 400x). Pyogenic granuloma showing an ulcerated surface covered by a fibrin membrane (D, HE 40x), and granulation tissue composed by infllammatory cells, small blood vessels and fibroblasts (E, HE 100x and F, HE 400x). Peripheral ossifying fibroma showing an ulcerated surface (G, HE 40x), and a proliferation of mesenchymal spindle cells associated with areas of calcified tissue (H, HE 100x and I, HE 400x). Peripheral giant cell lesion characterized by the presence of a vascularized tissue with deposition of hemosidherin (J, HE 40x), and details of the hemorrhagic areas associated with the presence of multinucleated giant cells and mononuclear cells (K, HE 100x and L, HE 400x).

Histological analysis showed that PG showed ulceration (*p*<0.001), were permeated by an acute inflammatory infiltrate (*p*<0.001), showed moderate/intense inflammatory infiltrate (*p*<0.001) and showed a loose connective tissue (*p*<0.001) more frequently than the other three groups. Red lesions were more frequently permeated by a moderate/intense inflammatory infiltrate, especially in FH and POF (*p*=0.002). Fibroblastic proliferation was more common in POF (*p*<0.001), while vascular proliferation was more common in PG (*p*<0.001). Mineralized material was more frequently encountered in POF (*p*<0.001), and multinucleated giant cells were found in all PGCL, but they have been also encountered with lower frequencies in the other three lesions (*p*<0.001). Hemorrhage was more common in PG and PGCL (*p*<0.001) and the presence of bacterial colonies was uncommon in all studied disorders (Table 2 and Fig. 3).

**Table 2:**
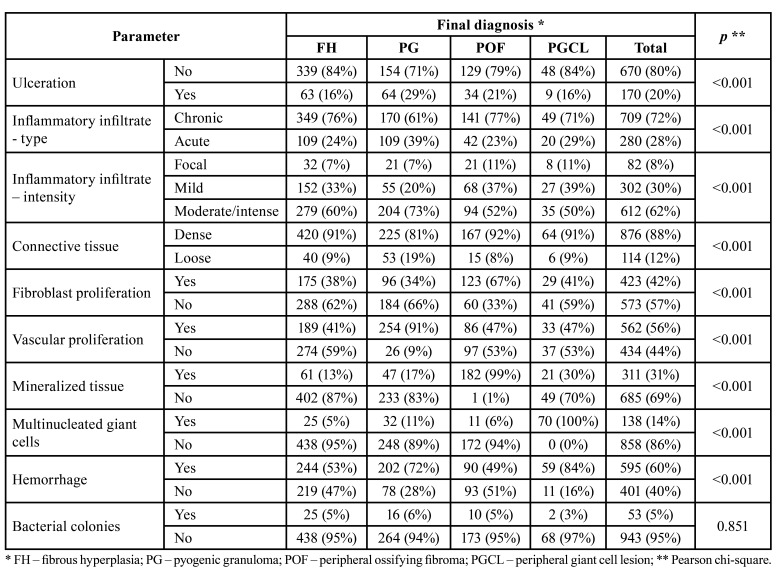
Distribution of the histological parameters by final diagnosis.

## Discussion

The present results showed that FH, PG, POF and PGCL are, in this order, the most common GRHL. Previous literature has shown that the frequency of each lesion may be variable, such as the FH counting from 13 to 68%, PG counting from 10 to 57%, POF counting from 4 to 39%, and PGCL counting from 1 to 47% (4-11,13-18) (Table 3). Most differences found in the frequency of these entities in different studies could be atribuTable to diagnostic criteria applied to histological analysis, availability of representative sections from each specimen and nomenclature and grouping of the conditions in specific categories. It is not possible, however, to rule out the possibility of genetic and, consequently, geographic differences on the frequency of these entities in different populations. Future prospective multicentric studies using the same methods are desired to address this topic.

As expected, the present results showed a high frequency of the most characteristic histological features from each condition, namely vascular proliferation for PG, mineralized material for POF and multinucleated giant cells for PGCL. All these features were, however, found in some lesions from other groups as well, reinforcing the evidences that there is some overlap in their histological diagnostic criteria. This could naturally justify some of the differences in frequency found in the literature, as previously stated, and also support the concept that these conditions are different tissue reactions to similar trigger factors (1-3).

Females are more affected by FH, PG and POF, as shown by most studies and by the present results (4-6,10-13,16,19-23). Few studies have shown an equal distribution or a male predominance for any of these three conditions (7,8,14,15). PGCL showed a more heterogeneous gender distribution, with studies showing female predominance (4,6,11,14-16,24), almost equal distribution (5,7,13,25) (also present results) or male predominance (10,26).

**Table 3:**
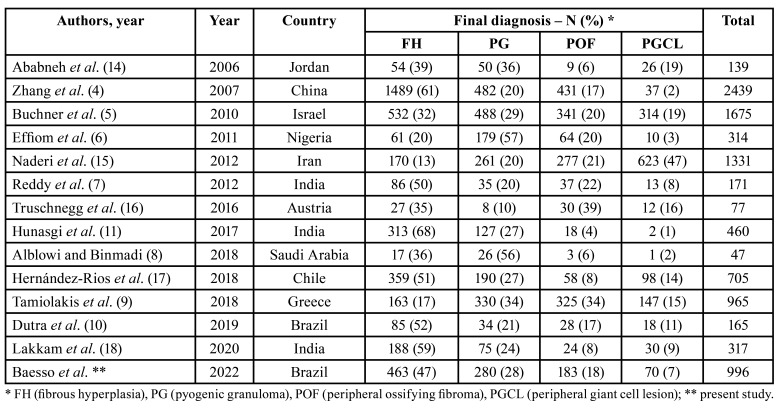
Frequency of the gingival/alveolar mucosal reactive hyperplastic lesions in the literature published in the last 15 years.

Curiously, all three studies derived from Brazilian populations, and also the present results, showed a slight or marked predilection of PGCL for males (10,26,27).

The present results showed that mean age of the patients affected by FH, PG and POF was in the fifth decade, and for PGCL in the sixth decade, at least in partial accordance with some studies (4,5,9,22,23). Most studies, however, have shown that mean age of the patients affected by these conditions is in the third to fourth decades of life (6-8,11,15,19-21,23). It has been suggested that POF is part of a maturation process in GRHL and that formation of mineralized material is a late event in their progression; mean age of POF affected patients is, however, similar or even lower than other conditions from this group not supporting this theory (11,15,28). POF is a relatively common GRHL in children and adolescents (29), and Buchner *et al*. (28) have shown that POF was the most common GRHL in patients under 19 years of age. This is, however, not shared by other studies, and Da Silva *et al*. (27), for example, have showed that PG was the most common GRHL in children up to 18 years of age.

The gingiva/alveolar mucosa of the upper anterior region is the most common anatomical location for FH, PG and POF, followed by the lower anterior region (6,9,11,19-21) (also present results). This predilection can be at least partially explained by the lower presence of saliva and also by the alterations in tooth positioning in this region, bringing difficulties to oral hygiene procedures and, consequently, facilitating the maintenance of local irritative factors (11). On the contrary, PGCL did not show a similar distribution pattern, and some studies have shown a predilection for the lower anterior region (24,25) or no clear predilection for the anterior or posterior regions and even for upper and lower gingiva/alveolar mucosa (4,5) (also present results). There is still no explanation for this anatomical difference in PGCL in comparison to FH, PG and POF, but they are probably related to different local factors that could determine specific tissues responses to irritative stimuli.

GRHL are, in general, mostly pedunculated, and, according with the present results, sessile lesions are most found in FH and PGCL. The revised laboratory charts did not include the clinical size of the lesions in most cases, so we have considered the size of the gross specimen derived from excisional biopsies as a reference for comparison of the size of the lesions among the three groups. PGCL represented the larger lesions in the present results, followed by POF. Apart from the differences in insertion on the adjacent tissues and size, most lesions in the present study were managed through conservative excisional biopsies, supporting that their clinical aspect was compatible with well-delimited benign conditions. It is also interesting to notice that most lesions showed, at least partially, some red areas compatible with the presence of the vascular inflammatory component, similarly to other studies (11). Time of onset showed that the lower values were associated with PG and we speculate that the clinical red aspect of the lesions and the presence of bleeding more commonly in this group could justify this difference. This would even be associated with the smaller size of the PG found in the present series, as patients tend to look after early profesional advice when lesions are symptomatic. Zhang *et al*. (4) have reported a mean time of onset for GRHL of 15 months (similar to the 13.4 months from the present series) and also showed lower mean time of onset for PG.

Histological analysis of the specimens showed that, as previously said, the presence of ulceration, presence of an acute inflammatory infiltrate, presence of moderate/intense inflammation, presence of a loose connective tissue, and presence of vascular proliferation were more common in PG. However, all these features were found in the other three groups, reinforcing the idea that some tissue responses are shared by all entities from these groups. PG are frequently ulcerated (19), but, for example, it has been reported that more than 50% of the POF can be also at least focally ulcerated, clinically simulating a PG (29). Mergoni *et al*. (22) have also shown that ulceration was present in 30% of the 27 POF included in their sample, and Reid Lester *et al*. (25) showed that 50% of the 279 PGCL included in their study presented ulceration. Salum *et al*. (26) also did not find differences in the frequency of ulceration when comparing PG, POF and PGCL.

POF were characterized by a proliferation of fibroblasts and the presence of mineralized material (23). The latter was not exclusively found in POF, and focal areas of mineralization were found in about 15% of the FH and PG and in 30% of the PGCL included in the present study. Hunasgi *et al*. (11), using a similar histological analysis than in the present study, showed that two out of 127 PG included in their series presented focal areas of bone neoformation. Reid Lester *et al*. (25) reported that 29% of the 279 PGCL included in their sample showed at least some areas of calcification, and 5% of the cases presented overlapping features with another condition, mostly POF. Multinucleated giant cells are essential for the diagnosis of PGCL, but they were also focally present in 5 to 10% of the other GRHL from the present sample. Contrarily, Hunasgi *et al*. (11) have encountered multinucleated giant cells only in PGCL.

It is important for Oral Medicine practitioners to keep in mind the clinical features that characterize each entity classified in the group of GRHL. It is also essential for Oral pathologists to take in account the heterogeneous histological features that can be encountered in these entities for proper diagnosis and to establish their most specific microscopic profile. As Periodontists are usually the first professionals in contact with patients presenting GRHL is of upmost relevance that this group should be familiarized with their clinical and histological profile.
